# Earth Without Life: A Systems Model of a Global Abiotic Nitrogen Cycle

**DOI:** 10.1089/ast.2017.1700

**Published:** 2018-07-01

**Authors:** Matthieu Laneuville, Masafumi Kameya, H. James Cleaves

**Affiliations:** ^1^ Earth-Life Science Institute, Tokyo Institute of Technology, Tokyo, Japan.; ^2^ The Institute for Advanced Study, Princeton, New Jersey, USA.; ^3^ Blue Marble Space Institute of Science, Washington, DC, USA.; ^4^ Center for Chemical Evolution, Georgia Institute of Technology, Atlanta, Georgia, USA.

**Keywords:** Nitrogen cycle, Abiotic, Planetology, Astrobiology

## Abstract

Nitrogen is the major component of Earth's atmosphere and plays important roles in biochemistry. Biological systems have evolved a variety of mechanisms for fixing and recycling environmental nitrogen sources, which links them tightly with terrestrial nitrogen reservoirs. However, prior to the emergence of biology, all nitrogen cycling was abiological, and this cycling may have set the stage for the origin of life. It is of interest to understand how nitrogen cycling would proceed on terrestrial planets with comparable geodynamic activity to Earth, but on which life does not arise. We constructed a kinetic mass-flux model of nitrogen cycling in its various major chemical forms (*e.g.,* N_2_, reduced (NH_*x*_) and oxidized (NO_*x*_) species) between major planetary reservoirs (the atmosphere, oceans, crust, and mantle) and included inputs from space. The total amount of nitrogen species that can be accommodated in each reservoir, and the ways in which fluxes and reservoir sizes may have changed over time in the absence of biology, are explored. Given a partition of volcanism between arc and hotspot types similar to the modern ones, our global nitrogen cycling model predicts a significant increase in oceanic nitrogen content over time, mostly as NH_*x*_, while atmospheric N_2_ content could be lower than today. The transport timescales between reservoirs are fast compared to the evolution of the environment; thus atmospheric composition is tightly linked to surface and interior processes.

## 1. Introduction

Atmospheres are the key observables that can be obtained remotely of planetary bodies within our solar system, as well as those orbiting other stars. While biosignatures are often studied to potentially detect biotic activity, relatively less research has explored how “geo”-signatures can improve our understanding of planets. In our solar system, for instance, Venus, Earth, and Mars have very different atmospheric nitrogen content, which may inform us about their history. In this contribution, we construct a planetary evolution model to track nitrogen distribution among major planetary reservoirs as mediated by abiotic processes known to occur on Earth. One goal of this study is to help understand what nitrogen content in planetary atmospheres can tell us about its evolution.

Nitrogen (also referred to as N for the sake of accounting for the mass of nitrogen atoms), in the form of N_2_ (or molecular nitrogen), is the major component of the present atmosphere and plays important roles in biochemistry (Thomazo and Papineau, 2013). Molecular nitrogen species in biological metabolism are transformed by anabolism, which endergonically synthesizes nitrogenous building blocks, and catabolism, which produces energy by converting high-energy molecules (*i.e.,* 19 NH_*x*_, NO_*x*_) into low-energy ones (N_2_). Today, bioavailable nitrogen (*i.e.,* all molecular forms except N_2_), together with iron and phosphorous, is often a growth-limiting nutrient in the environment (Smith, 1984; Vitousek and Howarth, 1991).

The timing of the origin of life is not well constrained, with some authors estimating that Earth may have become habitable by 4.4 Ga (Wilde *et al.,* 2001), there being some evidence of biology before 4 Ga (Dodd *et al.,* 2017) and stronger evidence for biology between ∼3.8 and 3.5 Ga, which remains actively debated (*e.g.,* Mojzsis *et al.,* 1996; Noffke *et al.,* 2013). Though there is some evidence that biological N_2_ fixation may have developed as early as 3.2 Ga (Stüeken *et al.,* 2015) and biological nitrogen utilization may have been active by 3.8 Ga (Papineau *et al.,* 2005), this still leaves potentially 0.5–1 billion years during which biology could have existed on Earth but had little effect on the drawdown of nitrogen from the atmosphere. Earth is sometimes assumed to have a chondritic composition (McDonough and Sun, 1995; Javoy *et al.,* 2010), although there are differing opinions (Campbell and O'Neill, 2012). Assuming either a carbonaceous or enstatite chondrite composition gives a range of values for the bulk terrestrial nitrogen inventory of 2.0–3.3 × 10^20^ kg (Johnson and Goldblatt, 2015). After the core (the N content of which is unknown), the major reservoirs are the atmosphere, the crust, sediments, and the mantle with on the order of 10^18^ kg in each reservoir, while the oceans only hold about 10^16^ kg (Table 1). The oceans, however, serve as a major conduit between the atmosphere and Earth's interior and therefore play an important role in the global nitrogen cycle. Similarly, biomass is a small reservoir (about 10^15^ kg), but the nitrogen flux through it is large. Although the core is potentially the largest nitrogen reservoir, it is ignored in this model: even if the extent of core-mantle interactions is debated, nitrogen fluxes between these two reservoirs are likely small (Hayden and Watson, 2007). More details about the constraints for the nitrogen content of each reservoir can be found in Appendix A.

**Table 1.** Estimates for the Sizes of Earth's Nitrogen Reservoirs

*Reservoir*	*Mass N*	*Major species*
Atmosphere	4.0 × 10^18^ kg	N_2_
Biomass	1.0 × 10^15^ kg	NH_*x*_
Oceans dissolved	2.4 × 10^16^ kg	N_2_
Sedimentary rocks	2 × 10^17^ kg	NH_*x*_
Continental crust	1.7 × 10^18^ kg	NH_*x*_
Mantle	2.8 × 10^19^ kg	NH_*x*_
Core	1.7 × 10^20^ kg	Fe_*x*_N_*y*_

Values are cited from the work of Johnson and Goldblatt (2015).

The way nitrogen cycles between reservoirs on terrestrial planets is contingent on a number of variables. For example, Venus, though slightly smaller than Earth, may have accreted from similar bodies to those that formed Earth and thus initially have had a proportionately similar amount of nitrogen (Wordsworth, 2016) (see Table 2). Although the proportion of nitrogen in Venus' atmosphere is smaller in terms of mass percent (3.5% vs. 78%), its total atmospheric N content is roughly 4 times that of Earth, further suggesting a large fraction of Earth's N is contained in deep reservoirs that have not outgassed or are replenished by subduction to a degree not possible on Venus (Wordsworth, 2016). The N content of Venus' atmosphere is roughly commensurate with an entire original chondritic inventory being present in its atmosphere.

**Table 2.** Measured and Estimated Abundances of Nitrogen in the Atmospheres and Interiors of Venus, Earth, and Mars

	*Venus*	*Earth*	*Mars*
Total surf. pressure (bar)	92	1	0.01
Atmospheric mass (kg)	4.8 × 10^20^	5.2 × 10^18^	2.5 × 10^16^
Atmospheric N mass (kg)	1.7 × 10^19^	4.0 × 10^18^	4.7 × 10^14^
Atmospheric N fraction	0.035	0.78	0.0189
Mass planet	4.9 × 10^24^	6.0 × 10^24^	6.4 × 10^23^
Est. bulk silicate N (kg)	2.2 × 10^19^	3.4 × 10^19^	2.9 × 10^18^
N atm./bulk silicate N	0.76	0.12	1.63 × 10^−4^

Data from http://nssdc.gsfc.nasa.gov/planetary/factsheet and Johnson and Goldblatt (2015). The bulk silicate N values were estimated by scaling Earth's value to Venus' and Mars' masses.

Likewise, Mars, though it has a tenuous atmosphere (mostly due to its small mass and lack of a geodynamo, which may have allowed widespread volatile loss over its history [Bogard *et al.,* 2001]), has a significant atmospheric nitrogen component (∼2 wt %, Mahaffy *et al.,* 2013). While deep martian nitrogen reservoirs remain almost wholly unknown (*e.g.,* Mohapatra and Murty, 2003), it has been estimated that Mars has lost 99% of its original atmospheric content (McElroy *et al.,* 1977; Hutchins and Jakosky, 1996). Beyond the N_2_ and NO*_x_* species present in its atmosphere, nitrates have now been detected on the surface (Stern *et al.,* 2015) and in martian meteorites (Kounaves *et al.,* 2014), suggesting there are some geochemical processes common to Earth and Mars such as fixation by meteorite impacts or lightning.

The evolutionary dynamics of Venus and Mars are not explored in this study, but we note that, once the influence of key processes on nitrogen distribution is identified, the different fates of nitrogen on the three bodies can be used to set constraints on their relative evolution.

The evolution of terrestrial nitrogen cycling has been studied previously, with different goals than those explored here. Mancinelli and McKay (1988) used constraints on abiotic fixation mechanisms to discuss the evolutionary sequence of biological nitrogen metabolism. In particular, they provided improved lightning and shock-induced fixation rates to argue that nitrogen fixation evolved later than denitrification, due to the relative availability of fixed nitrogen in Earth's early environment.

Later, Zhang and Zindler (1993) presented a global cycling model using new constraints on mantle degassing rates from CO_2_/He and N_2_/Ar ratios at mid-ocean ridges. They used a 2-box model to track the amount of “degassable” carbon and nitrogen as a function of time and therefore neglected any flux buffering associated with nitrogen fixation. They found an almost constant atmospheric N_2_ pressure after an initial transient stage of several 100 Ma, and that long-term variation in recycling efficiency would not produce observable effects on atmospheric measurements because of the timescales involved.

More recently, Stüeken *et al.* (2016) developed an updated evolutionary model focusing on atmospheric nitrogen content to test whether abiotic processes could be the cause of the potentially large fluctuations in pN_2_ observed (Marty *et al.,* 2013; Som *et al.,* 2016). They constructed a box model with imposed global variations (such as global oxygenation events, global crustal melting) and tested how they propagate through the system. Long-term variation in atmospheric nitrogen content was found to be below observable levels, suggesting a biotic origin for the large fluctuations in pN_2_.

In our model, we consider speciation of nitrogen (N_2_, reduced and oxidized) and further keep the ocean as a separate reservoir, specifically the only reservoir where the different nitrogen species can coexist in significant amounts. As different redox state nitrogen species react differently in the environment (*e.g.,* only reduced nitrogen is efficiently adsorbed onto sediments), bottlenecks may be created in the system. In particular, we would like to understand how environmental variables affect global circulation of nitrogen.

In the next section, we present our geodynamical model and describe how the different nitrogen fluxes can be parameterized. We then present the predictions that can be made for an “Earth-like” body (meaning a body where the geodynamics and initial nitrogen budget are similar to those of Earth). We finally discuss which parts of this model most influence the ultimate planetary distribution of N and what inferences can be made regarding other bodies in our solar system and beyond.

## 2. Methods

**FIG. 1.**
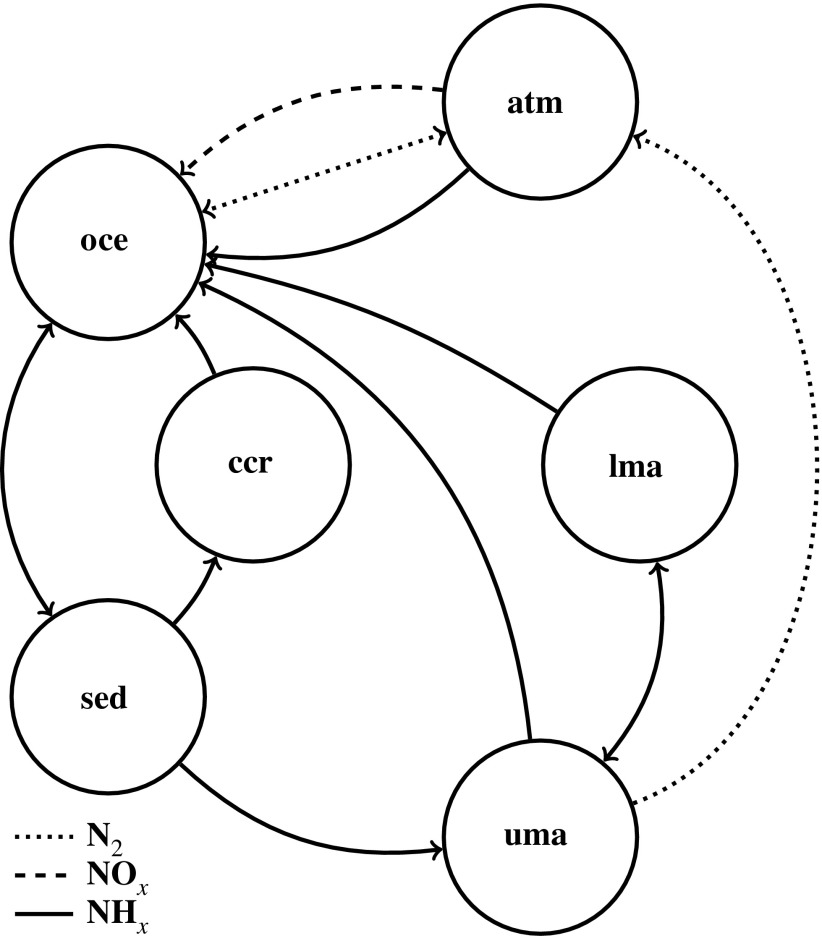
Model topology used in this study. Circles represent nitrogen reservoirs and correspond, clockwise, to atmosphere (atm), lower (lma) and upper (uma) mantle, marine sediments (sed), continental crust (ccr), and oceans (oce). Arrows represent fluxes between reservoirs, with arrow style indicating the nitrogen speciation of that flux.

Our basic model considers coupling between various reservoirs, where the mass of any species in a reservoir is a balance of the mass flux into the reservoir minus the flux out of the reservoir due to mass transfer and chemical reactions. We also consider speciation within reservoirs with respect to the redox state of nitrogen. Figure 1 presents a simplified model topology. In the following subsections, the detailed contributions of each flux are outlined.

The major nonbiological mechanisms that govern the flux of nitrogen on planetary scales are atmospheric fixation by impacts and lightning, rainout, aqueous phase chemistry, adsorption to mineral phases, subduction, and remineralization. The model presented here includes terms for reversible transport between the atmosphere and oceans, reactions in the aqueous phase, transformation in hydrothermal settings, reversible adsorption to mineral phases (*e.g.,* marine sediments), subduction of marine sediments, accretion of continental crust and erosion, transformation and outgassing during subduction, and release of mantle nitrogen via hotspot volcanism.

The continental crust volume as a function of time can be estimated from variation in the isotopic composition of zircons. Dhuime *et al.* (2012) identified a transition between an early rapid growth of the continents at about 3 km^3^ yr^−1^ until ∼3 Ga, followed by a longer, slower growth period at 0.8 km^3^ yr^−1^ from then until the present day. They speculate that this transition may be due to the onset of subduction, which reduced the effective amount of crust accreted onto continents. As such scenarios are still debated, we also tested a constant growth rate model (*e.g.,* Belousova *et al.,* 2010).

The values used in this model were compiled from careful consideration of likely sources and sinks of N species according to presently measured parameters and fluxes, but the error in these nitrogen mass flux estimates may be large (see the following subsections for more details). Below, we present the rationale for our choices. The variability of our conclusions and their dependence on uncertainties in the model parameters are discussed in the following section.

### 2.1. Extraterrestrial input

**FIG. 2.**
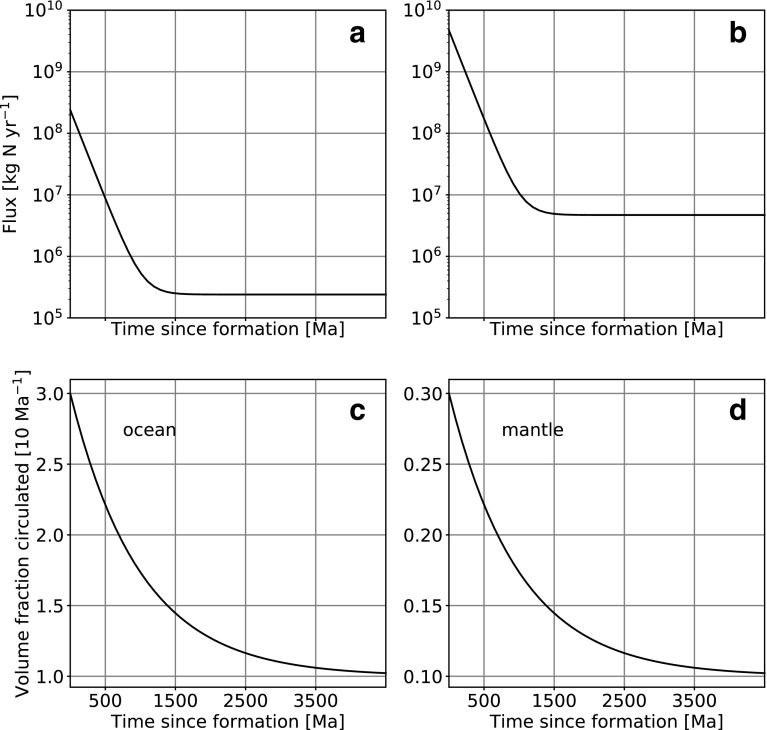
Model time dependence of the fluxes. (**a**) Net nitrogen addition to the Earth system from extraterrestrial sources. (**b**) Atmospheric N_2_ fixation by impacts. This flux is scaled to 1 PAL N_2_. (**c**) Ocean volume fraction circulated through hydrothermal vents per 10 Ma. (**d**) Mantle fraction circulated per 10 Ma. This constrains the rate at which the upper and lower mantle equilibrate. The last two functions act as a prefactor to the overall mass flux calculation.

Contributions from space are modeled as coming from two possible sources. The first is net input of reduced nitrogen from comets, asteroids, meteorites, and interstellar dust particles as described by Chyba and Sagan (1992). These calculations are based on cometary studies and the lunar cratering record, which have been used to determine the carbon fluxes from these sources. We used a N/C ratio in comets of 0.1 (Mumma and Charnley, 2011) to obtain a net nitrogen flux. However, Marty *et al.* (2016) suggested that cometary volatiles may have made a minor contribution to Earth's budget, so this should be considered a generous estimate. Even so, it can be seen that this contributes a relatively small amount of nitrogen to the modeled system (see Fig. 2a).

The second extraterrestrial contribution comes from impacts, which also likely served as an energy source to fix atmospheric N_2_ to soluble species. For impact shocks, the amount of endogenous carbon that gets fixed as a function of time has been computed (Chyba and Sagan, 1992). We assume here that nitrogen is fixed at the same rate as carbon. Depending on the redox state of the atmosphere, nitrogen abiotically fixed that way can be in the form of either reduced or oxidized compounds. In both cases, rainout time is considered to be negligible compared to geological timescales, and nitrogen was added as dissolved species to the ocean reservoir directly. The time dependence of this flux was parameterized as

\begin{align*}
F \left( t \right) = {F_0} + \left( {{F_1} - {F_0}} \right) \;{ \rm{ ex}}{{ \rm{p}}^{ - t / \tau }} \tag{1}
\end{align*}

where *τ* is 150 Ma and *F*_0_ and *F*_1_ are 2.4 × 10^5^ and 2.4 × 10^8^ kg N yr^−1^ for the cometary input and 4.7 × 10^6^ and 4.7 × 10^9^ kg N yr^−1^ for impact fixation, respectively. For impact fixation, the flux was linearly scaled to the present-day atmospheric nitrogen content of 4 × 10^18^ kg (*i.e., F* is divided by two if pN_2_ is 0.5 PAL). Figures 2a and 2b show the fixation time dependence for these fluxes. Note that impact statistics in the work of Chyba and Sagan (1992) is consistent with the concept of Late Heavy Bombardment but does not consider singular events such as the putative Moon-forming giant impact.

### 2.2. Atmosphere to surface

Yung and McElroy (1979) estimated abiotic nitrogen fixation due to atmospheric electric discharges as 1–4 × 10^10^ kg yr^−1^ in an oxidizing atmosphere and 2 × 10^9^ kg yr^−1^ in a reducing one. Borucki and Chameides (1984) gave the present rate of abiological atmospheric fixation as ∼2.6 × 10^9^ kg yr^−1^. Navarro-Gonzalez *et al.* (2001) showed experimentally that this value may be relevant for a CO_2_-dominated atmosphere, while the fixation rate in an N_2_-dominated atmosphere could be much lower, around 2.6 × 10^6^ kg yr^−1^. Galloway *et al.* (2013) also tabulated modern N fixation rates by lightning as between 3 × 10^9^ and 10 × 10^9^ kg N yr^−1^. For consideration, in the absence of remineralization processes (and assuming the rates are pressure-independent), these fixation rates would allow for the complete fixation of the present mass of atmospheric N_2_ in ∼0.1–1 Ga.

In general, the residence times of odd nitrogen species in the atmosphere, which include NH_3_, NO, NO_2_, HNO_3_, N_2_O, HONO_*x*_, and N-containing organic compounds, with the exception of N_2_O, are only hours to days (Chameides, 1975), which is much smaller than the time step in our model (5000 years). We therefore assume fixed nitrogen species are instantaneously transported to, and dissolved in, the oceans.

To account for abiotic fixation by lightning, we used a constant flux of N_2_ to NO*_x_* and NH_*x*_, linearly scaled to atmospheric pN_2_. For an atmospheric mass of 4 × 10^18^ kg, the fluxes to NO*_x_* and NH*_x_* are 4 × 10^10^ and 1.6 × 10^7^ kg N yr^−1^, respectively, for the CO_2_-dominated case and 2.6 × 10^6^ and 10^3^ kg N yr^−1^ for the N_2_-dominated one.

We used Henry's law to determine the equilibrium concentration of N_2_ in the oceans:

\begin{align*}
K = \frac { p }  { c } \tag { 2 } 
\end{align*}

where *K* is Henry volatility for nitrogen and *p* is N_2_ partial pressure in the atmosphere and *c* its concentration in the ocean. Assuming a modern average circulation time of 1600 years, the oceans are considered here as well mixed at all times. The timescales of soluble species reactivity and adsorption are also short relative to advection and diffusion; thus all species dissolved in the ocean reservoir also equilibrate instantaneously. Assuming a mean ocean temperature of 25°C, the Henry's law constant for N_2_ is 1600 L atm mol^−1^ (Sander, 2015), though this decreases by a factor of about 4 between 0°C and 100°C, which in turn would decrease the ratio of dissolved N_2_ to atmospheric N_2_ by a factor of 4 were the oceans at a mean temperature of 100°C.

We considered two atmospheric redox states: a generally neutral (N_2_-dominated) atmosphere and one with significant amounts of reduced gases (*e.g.,* CH_4_ or CO), corresponding to a low and high abiotic fixation rate, respectively. Both types are plausibly concordant with the geological record and planetary evolution models (Kasting *et al.,* 1993; Canil, 1997; Delano, 2001; Trail *et al.,* 2011). Though it seems possible that the upper mantle was more reduced during the earliest stages of Earth's differentiation (Arculus and Delano, 1980; Holland, 1984; Frost and McCammon, 2008), this is generally before the time period considered here. We also neglect the influence this parameter would have on the abiotic fixation rate, as the effect is not well parameterized.

### 2.3. Hydrothermal vent transformations

Presently, the entire ocean volume cycles through marine hydrothermal systems approximately every 10 million years (Holland, 1984), where a fraction of that volume then reacts within the vent systems. A higher rate of internal heat production on the early Earth would have led to increased volcanic and tectonic activity and a likely concurrent increase in hydrothermal vent activity and ocean circulation rates through them (Turcotte, 1980). We therefore explored system evolution with either constant or exponentially decreasing rates of hydrothermal circulation through time. We use the same functional form as Eq. 1, with *F*_0_ = 1 × 10^−7^ and *F*_1_ = 3 × 10^−7^ yr^−1^, respectively. The resulting evolution can be seen in Fig. 2c.

We assumed that the nitrogen-reduction reactions described by Brandes *et al.* (1998) (which describes the reduction of nitrite, nitrate, and N_2_ to NH_3_) and Summers (2005) (which describes the reduction of nitrite and nitrate to NH_3_ at lower temperatures than those of Brandes *et al.* [1998]) operate during hydrothermal circulation. Thus, all of the circulated NO*_x_* and 0.1% of dissolved N_2_ are converted to NH*_x_* via combined on- and off-axis alteration. The above-cited experiments have shown that nitrate and nitrite are reduced much more efficiently than N_2_; thus this ratio represents a plausible but necessarily arbitrarily low value as N_2_ reduction rates are not well constrained.

### 2.4. Marine sediment adsorption

To model NH*_x_* adsorption onto sediments, we used a Freundlich isotherm model, which relates the concentration of surface-adsorbed species to the concentration of dissolved species (Mackin and Aller, 1984):

\begin{align*}
{Q_{ \rm{e}}} = {K_{ \rm{f}}}C_{ \rm{e}}^{{1 / n}} \tag{3}
\end{align*}

where *Q*_e_ is the amount of solute adsorbed per mass of sediment at equilibrium (in mol g^−1^), *C*_e_ is the equilibrium solution concentration (in m*M*), *K*_f_ is an indicator of adsorption capacity, which depends on factors such as the number of available exchange sites per unit sediment mass or volume (the larger the value of *K*_f_, the more NH*_x_* can be adsorbed), and *n* is the Freundlich coefficient, which is an indicator of the strength of adsorption.

Generally *K*_f_ < 1, and *n <* 1 (where *n* is small, adsorption is strong). Both *n* and *K*_f_ are system-specific constants. We used values of *n* ∼ 1 and *K*_f_ ∼ 10^−3^ L/g, derived from the work of Mackin and Aller (1984). Although mean ocean temperature as a function of time remains uncertain, the temperature dependence of NH*_x_* adsorption on marine sediments has been found to be negligible over the range of 6–26°C (Mackin and Aller, 1984); thus a temperature term was not included in the model.

### 2.5. Subduction and continental erosion

The subduction flux of nitrogen may have changed over Earth's history, depending on subduction rates and the abundance of N in subducted sediments, but remains poorly constrained. Substantial amounts of nitrogen may be cycled into the deep mantle. It has been estimated that presently ∼3–6 × 10^10^ mol N yr^−1^ (4.2–8.4 × 10^8^ kg N yr^−1^) is transported to the mantle by cold slabs (Busigny *et al.,* 2003; Goldblatt *et al.,* 2009).

While there may be a significant amount of nitrogen in sediment pore water, at the current rate of about 1 km^3^ yr^−1^, it would take about 1.4 billion years to cycle the entire ocean volume through the world's accretionary prisms (Huene and Scholl, 1991). This is about 2 orders of magnitude slower than the present cycling time of the oceans through hydrothermal vents at mid-ocean ridges. Thus, the chemical processes associated with hydrothermal circulation at mid-ocean ridges likely have a larger effect on ocean N chemistry than do the low-temperature waters expelled from accretionary prisms.

The modern oceanic crust is recycled on average every 100 Ma (*e.g.,* Müller *et al.,* 2008), subducting ∼1.34 × 10^7^ to 2.1 × 10^10^ kg N yr^−1^. For the base scenario, we assume a constant subduction rate with a timescale of 100 Ma for complete oceanic crust turnover. To consider the addition of nitrogen to the continental crust, we used a constant accretion efficiency factor *ε* which governs the fraction of the nitrogen subduction rate which is accreted to the continental mass (independent of the continental growth model). As this value is poorly constrained, and in order to retain the ability to draw general inferences using the model, we varied this parameter over the entire range from 0 to 1:

\begin{align*}
 { F_ { { \rm { sed \hbox {-} uma } } } } = ( 1 - \epsilon ) \frac { { { M_ { { \rm { sed } } } } } }  { \tau } \tag { 4 } 
\end{align*}

\begin{align*}
 { F_ { { \rm { sed \hbox {-} ccr } } } } = \epsilon \frac { { { M_ { { \rm { sed } } } } } }  { \tau } \tag { 5 } 
\end{align*}

where *M*_sed_ is the mass of nitrogen in the sediments and *τ* the subduction rate.

Finally, continental nitrogen can be returned to the oceans by erosion. Estimates for modern continental erosion rates vary from 10^−5^ to 5 × 10^−5^ m yr^−1^ (Von Blanckenburg, 2005), with the total flux proportional to the continental surface area. By multiplying this value by the surface area of the continental crust as a function of time, we can compute the return flow of nitrogen species to the oceans.

### 2.6. Mantle to atmosphere

The present flux of nitrogen from Earth's mantle to the atmosphere has been variously estimated as 2.8–30 × 10^9^ mol N_2_ yr^−1^ (7.84–42 × 10^7^ kg N yr^−1^) (Marty, 1995; Sano *et al.,* 2001). About ∼25 km^3^ yr^−1^ of upper mantle material surfaces as magma, which is distributed as follows: 19% (∼5 km^3^) in arcs (with the majority of the N released as N_2_) and 81% (∼20 km^3^) in mid-ocean ridge basalt (MORB) (with the majority of the N released as NH_*x*_) (Coffin *et al.,* 2002). From the lower mantle, ∼2.5 km yr^−1^ comes out in hot-spot volcanism, and we assume all of the N outgassed by this mechanism is NH_*x*_. It is worth noting that, given the present subduction estimates indicated in Section 2.5, the net flux of nitrogen between Earth's surface and interior could be in either direction.

The present-day nitrogen distribution and flux values are assumed to be the outcome of both the nitrogen content and redox state of the mantle. We linearly scaled the bulk volcanic output in the model to the mantle nitrogen content divided by the present-day value as a reference. Mikhail and Sverjensky (2014) showed that, in aqueous fluids at conditions prevailing in Earth's upper mantle, nitrogen can be present either as N_2_ or NH_*x*_. Further, Li *et al.* (2015) also showed that nitrogen speciation in silicate melts strongly depends on oxygen fugacity. To asses the importance of this partitioning, we therefore varied the partition of upper mantle degassing between reduced species (MORB-like) and neutral (arc-like) with a redox parameter *α*_m_, which is 0.19 for the present mantle (Sano *et al.,* 2001). We did not consider the time dependence of this parameter in order to avoid extra complexity and simply analyzed a range of constant values. We also neglected how redox state would influence rock mineralogy and therefore the preferential retention of NH*_x_* in the mantle.

A typical convection speed in the present-day mantle is on the order of 1–10 cm yr^−1^, leading to mixing rates on the order of 10^−9^ to 10^−8^ yr^−1^ (Ferrachat and Ricard, 1998; Van Keken and Zhong, 1999). There is significant uncertainty in these estimates, mostly due to unknown thermophysical properties at high-pressure, high-temperature conditions and potential chemical layering (Van Keken and Zhong, 1999); we therefore used values within this range as an estimate. We expect convection to be more vigorous early in Earth's history; thus we scaled mantle mixing as a decreasing exponential, as was done for ocean circulation. For this time dependence, we used *F*_0_ = 10^−8^, *F*_1_ = 3 × 10^−8^ yr^−1^, and *τ* = 10^9^ yr. The model evolution can be seen in Fig. 2d. We also considered cases with constant mixing rates to evaluate the importance of this parameter.

## 3. Results

In this section, we present the implications of nitrogen cycling on a planet with similar geodynamical processes as the modern Earth. First, we show the evolution resulting from “best guess” model parameters, as presented in the previous section, which includes an atmosphere with a high abiotic fixation rate and a mantle redox state similar to the present day, with evolving dependence on mantle mixing and ocean hydrothermal circulation rates. Then, we show the influence of those rates, initial conditions, and mantle redox state on the nitrogen content of the atmosphere and volcanic degassing fluxes.

### 3.1. Representative evolution

**FIG. 3.**
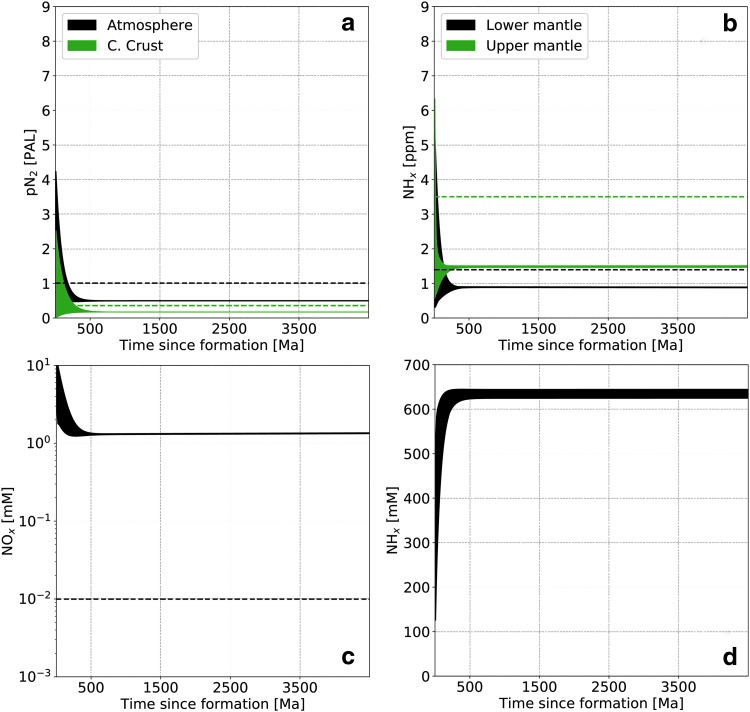
Nitrogen content as a function of time in the main reservoirs for a high abiotic atmospheric fixation rate. (**a**) Atmospheric N_2_, (**b**) upper and lower mantle (NH_*x*_), dissolved oceanic NO*_x_* (**c**) and NH*_x_* (**d**). Dashed lines represent present-day values. For oceanic NH*_x_* this value is 3 × 10^−4^ m*M* and therefore does not appear on the figure. The randomly seeded range of initial conditions all collapse on the same evolutionary trend during the first ∼500 Ma (see also Fig. 5).

**FIG. 4.**
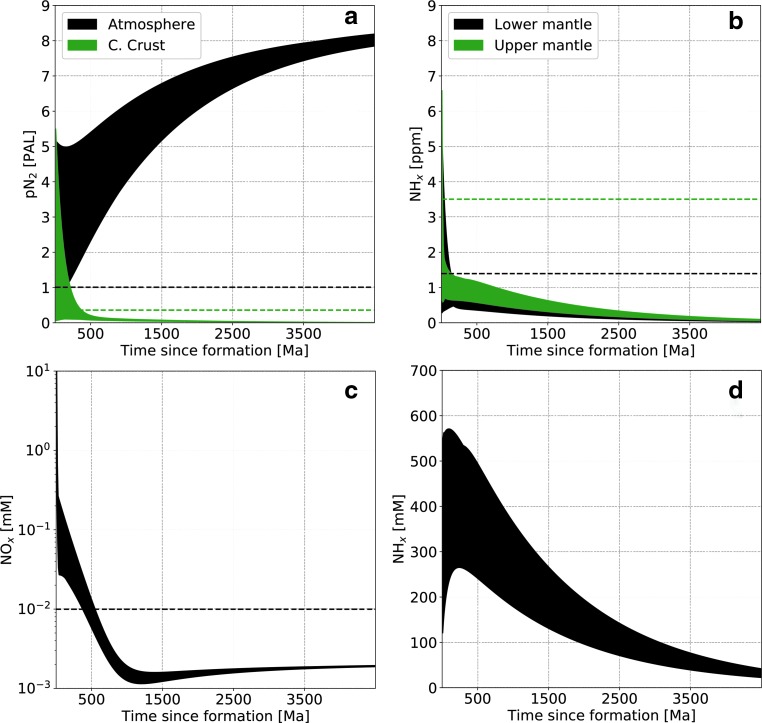
Nitrogen content as a function of time in the main reservoirs for a low abiotic fixation rate. (**a**) Atmospheric N_2_, (**b**) upper and lower mantle (NH_*x*_), dissolved oceanic NO*_x_* (**c**) and NH*_x_* (**d**). Dashed lines represent present-day values. For oceanic NH*_x_* this value is 3 × 10^−4^ m*M* and therefore does not appear on the figure. The randomly seeded range of initial conditions take several billion years to collapse on the same evolutionary trend (see also Fig. 5).

A typical simulation, with parameters described in Table 3, leads to the system evolution shown in Fig. 3 (high abiotic atmospheric fixation rate) and Fig. 4 (low abiotic atmospheric fixation rate). We start with a random distribution of initial conditions in phase space and integrate forward in time. Present terrestrial values are shown as dashed lines and correspond to a bulk silicate Earth content of 3.4 × 10^19^ kg N (*i.e.,* including all reservoirs except the core), as described in Table 1.

**Table 3.** Main Simulation Parameters and Their Default Values

*Name*	*Parameter*	*Default value*
Mantle redox state	*α*_m_	0.2
Atmosphere redox state	*α*_a_	0.2
Subduction rate	*D*	100 Ma
Continental accretion	*ε*	0.05
Erosion rate	—	5 × 10^−4^ m yr^−1^
Ocean hydrothermal circulation		
Decay constant	*τ*	150 Ma
Min value	*F*_0_	2.4 × 10^5^ kg N yr^−1^
Max value	*F*_1_	2.4 × 10^8^ kg N yr^−1^
Mantle mixing rate		
Decay constant	*τ*	10^9^ yr
Min value	*F*_0_	1 × 10^−8^ yr^−1^
Max value	*F*_1_	3 × 10^−8^ yr^−1^

The main difference with the present-day Earth is the much larger nitrogen content in the oceans, mostly present as NH_*x*_. As a consequence, the mantle and continental crust have lower nitrogen content than the modern Earth. It is interesting to note here that the ocean is the only reservoir that hosts all three nitrogen speciation types in abundance and therefore serves as a switchboard for the global balance. In the low abiotic fixation rate (Fig. 4), nitrogen is not fixed from the atmosphere robustly and therefore reaches several present atmospheric levels (PAL), leaving other reservoirs with much lower concentrations. Note that we do not plot the marine sediments reservoir content evolution as it is directly related to the oceanic NH*_x_* concentration according to Eq. 3, but it can hold a few PAL nitrogen, as shown in Table 4.

**Table 4.** Average Mass Balance Corresponding to Figures 3 and 4

*Reservoir*	*Figure 3*	*Figure 4*
Atmosphere	0.50	8.00
Continental crust	0.17	0.01
Marine sediments	3.05	0.14
Oceans N_2_	0.00	0.04
Oceans NO_*x*_	0.02	0.00
Oceans NH_*x*_	3.82	0.18
Upper mantle	0.36	0.02
Lower mantle	0.56	0.03

All values are in PAL.

Two evolutionary timescales can be observed. The first characterizes the time required for the system to “forget” its initial conditions, which is about 500 Ma in Fig. 3 and several billion years in Fig. 4. The second is a long-term system evolution governed by environmental properties. This can be understood in terms of approach to steady state and the evolution of the steady state itself. In the high abiotic fixation rate case, only a few hundred million years are necessary to reach the steady state defined by the model parameters from a wide range of initial conditions. Then, as ocean and mantle mixing rates and the impact flux decrease with time (see Fig. 2), the steady state varies and is tracked by the actual state of the system (*i.e.,* the long-term evolution). This is more visible in the high abiotic atmospheric fixation rate case (Fig. 3) because of the faster cycling timescales throughout the network, but initial condition collapse also happens, although less dramatically, in the low abiotic atmospheric fixation case (Fig. 4).

**FIG. 5.**
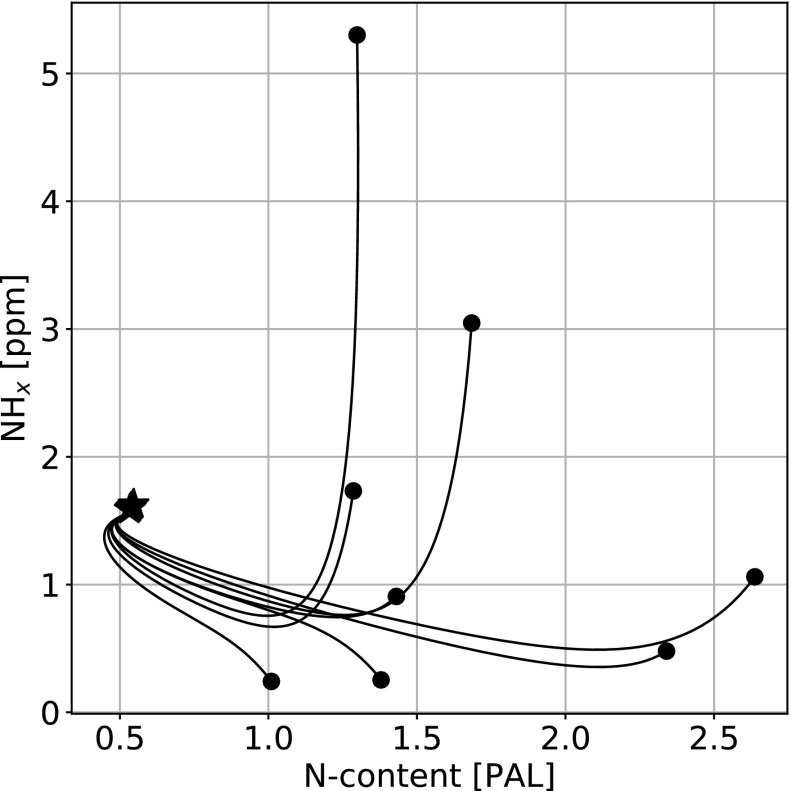
Example of evolutionary curves in the atmospheric N_2_ (*x* axis)/upper mantle NH*_x_* (*y* axis) plane for the model presented in Fig. 3. Circles denote initial conditions and the star the common final state. Every simulation joins the common evolutionary track in less than 500 Ma.

Figure 5 shows a random selection of trajectories in the phase space between atmospheric N_2_ (*x* axis) and upper mantle NH*_x_* (*y* axis) for the high abiotic fixation case. As suggested in Figs. 3–4, irrespective of the initial conditions, the nitrogen distribution is attracted to a steady state determined by the system parameters (upper mantle redox state, ocean hydrothermal circulation, and mantle mixing rates, etc.).

Our initial conditions are randomly distributed in phase space; therefore early evolution corresponds to the relaxation of those initial conditions on a self-consistent track for a given set of model parameters. Interestingly, this common evolutionary track is reached quickly, allowing for major distribution changes early in system evolution.

### 3.2. Parameter sensitivity

**FIG. 6.**
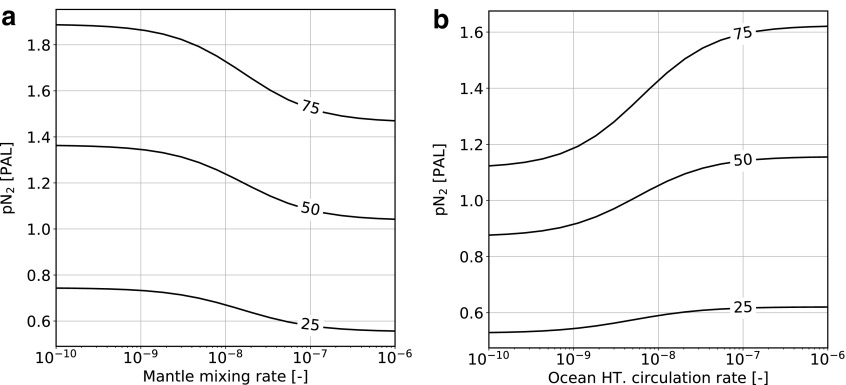
Atmospheric nitrogen content after 4.5 Ga as a function of (**a**) mantle mixing rate and (**b**) ocean hydrothermal circulation rate for different values of *α*_m_ in the high abiotic atmospheric fixation rate case. The parameter *α*_m_ controls the partitioning between different volcanic degassing styles; see the text for more details.

Figure 6 shows the steady state pN_2_ as a function of mantle mixing and ocean hydrothermal circulation rates for the high abiotic atmospheric fixation rate scenario. The two limiting cases of “no mixing” and “perfect mixing” can be seen as the asymptotes of the different curves (note that the two parameters were varied independently here). A low mantle convection rate favors a larger atmospheric N_2_ content as more nitrogen is degassed through arc systems as N_2_. Conversely, more effective mantle mixing increases the fraction of nitrogen degassed by hot-spot volcanism, which is more reduced (and therefore eventually dissolves as oceanic NH_*x*_).

The ocean hydrothermal circulation rate controls the fraction of ocean water that is circulated through hydrothermal vents per year. A high circulation rate favors efficient circulation of nitrogen between the different reservoirs, as oceanic nitrogen is readily reduced and subducted. The outcome is a larger atmospheric N_2_ content. Low values, however, prevent effective burial to the mantle and therefore also imply a lower degassing rate to the atmosphere.

**FIG. 7.**
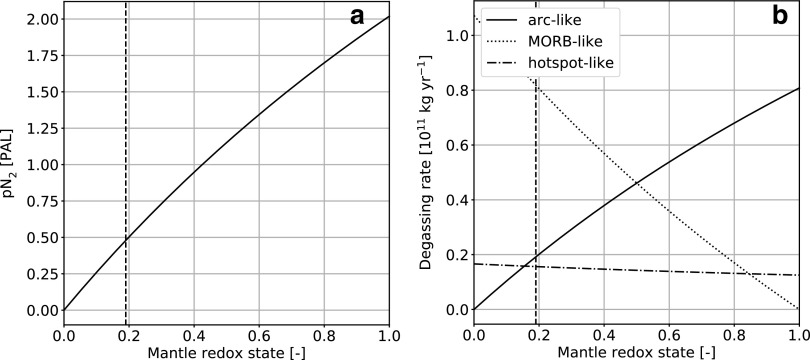
(**a**) Atmospheric nitrogen content and (**b**) volcanic degassing rate as a function of mantle redox state *α*_m_ after 4.5 Ga for the high abiotic atmospheric fixation rate case. The redox state controls the relative contribution of arc-like (high values, degassed as N_2_ to the atmosphere) to MORB-like (low values, degassed as NH_*x*_, which rains out instantaneously to the oceans) volcanic degassing. The vertical dashed line represents the value estimated for present-day Earth.

We can now examine how the steady state nitrogen distribution between various reservoirs varies with the redox state of the upper mantle and atmosphere. Increasing the mantle redox state (Fig. 7) controls the fraction of mantle nitrogen released by volcanism as N_2_ (arc-like) versus NH*_x_* (MORB-like). Figure 7 shows that, when the mantle is more oxidized, the atmospheric N_2_ content increases, which subsequently forces an increase in both oceanic dissolved N_2_ (through Henry's law) and NO*_x_* (through abiotic fixation). This confirms that upper mantle redox state, or more specifically the partitioning of volcanic gases between reduced and oxidized species, can control the long-term state (pressure and composition) of the atmosphere. Note here that *α*_m_ cannot be directly related to redox buffers such as fayalite-magnetite-quartz as it is only an effective partitioning between volcanic degassing fluxes, although this could be modeled separately.

**FIG. 8.**
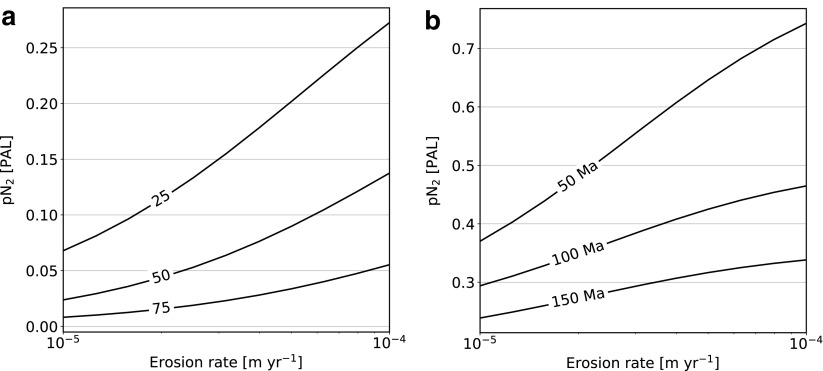
Atmospheric nitrogen content after 4.5 Ga as a function of erosion rate for different values of (**a**) continental accretion efficiency *ε* and (**b**) oceanic crust subduction timescale (parameter *D* from Table 3).

The oceanic crust subduction timescale and continental crust erosion timescale both play a large role in determining the final distribution of nitrogen between the existing reservoirs. Figure 8 shows the atmospheric nitrogen content at the present day as a function of erosion rate for various accretion efficiencies (left) and subduction timescales (right). Higher erosion rates always increase the amount of atmospheric nitrogen as it prevents efficient storage of nitrogen in the continental crust. Lower accretion efficiency has the same effect on nitrogen distribution. Irrespective of accretion efficiency, faster subduction decreases the average residence time of nitrogen in the oceanic crust and also leads to higher atmospheric nitrogen content.

The main result of this study is that, given a high abiotic fixation rate and a partition of volcanism type similar to today's (*i.e.,* 19% degassing from the upper mantle as N_2_ in arc-type volcanism, with the remaining 81% degassed as NH*_x_* from MORB-like volcanism, and all hot-spot-like volcanism from the lower mantle reduced), our global nitrogen cycling model predicts a significant increase in oceanic N content over time, while atmospheric N_2_ content remains lower than today. The situation is different in the low abiotic fixation case. In that scenario, the previous observations still hold; but the atmosphere also acts as a bottleneck, and the initial oceanic NH*_x_* reservoir is slowly transferred back to N_2_ in the atmosphere, where no process is able to mobilize it fast enough to prevent buildup. Another worthwhile observation is that the residence time of nitrogen in most reservoirs is small compared to geological times; therefore we expect large variations during early planetary evolution, as the system reaches steady state. In the next section we discuss the interpretation of these results and important limitations of the model.

## 4. Discussion

There are important caveats to be aware of before interpreting the outcome of this model. We present the major ones below but stress again what this model attempts to achieve. We chose a selection of fluxes and respective parameterizations that best match our understanding of present-day terrestrial abiotic nitrogen fluxes. The model is then integrated forward in time from planetary differentiation to the present day to obtain insight into what such processes imply for nitrogen distribution. Using this result to describe an “abiotic Earth” is more difficult, and we can only claim to model nitrogen cycling on a planet with similar geodynamical features as Earth.

### 4.1. Limits of the model

To that end, several simplifying assumptions had to be made. This model does not specifically take into account chemical reactions between gas-phase N-containing species and other atmospheric species such as C-containing gases (*e.g.,* CO_2_, CO, and CH_4_), though these could have a marked impact on some of the fluxes. For example, the oxidation state and pressure of C-containing gases govern the nature of N transformations in the atmosphere (a CH_4_-dominated atmosphere is prone to generating HCN and NH_3_, while a CO_2_-dominated atmosphere tends to predominantly generate NO*_x_* species [Folsome *et al.,* 1981; Heinrich *et al.*, 2007; Cleaves *et al.,* 2008]).

High pressures of CO_2_ can also have major effects on temperature and seawater pH, as well as Henry's law constants for the partitioning of atmospheric N-species. For N_2_ and NO*_x_* species this is likely not problematic, as N_2_ does not ionize in water and nitrite and nitrate are highly soluble across all reasonable pH values.

This model assumes that surface temperatures allow liquid oceans to exist, which are not predominantly ice covered. This is important with regard to N speciation as so many of the higher- and lower-oxidation-state species of N are water soluble. Global N-cycling cannot be entirely decoupled from the global hydrologic cycle (Barron *et al.,* 1989), which will be investigated in a subsequent study.

Other potentially important nitrogen fixation mechanisms (in this case yielding HCN, which hydrolyzes ultimately to formic acid and ammonia [Miyakawa *et al.,* 2002]) are extreme-UV solar radiation (Zahnle, 1986) and coronal mass ejections from the young Sun (Airapetian *et al.,* 2016). They are both estimated to be more efficient in a reducing atmosphere and may have helped fix a large fraction of early atmospheric N_2_. We did not model these processes, but we have shown that our results do not greatly depend on initial conditions; therefore they may be neglected over the long term. However, these processes may be important when considering the early availability of nitrogen for potential biotic processes.

The volume of the oceans is thought to have changed over time but was kept constant in our base simulations. It has been variously estimated that the volume of the oceans has decreased by as much as 60% since their formation (Korenaga, 2008), though other authors have suggested a general increase in their volume over the same time (Harrison, 1999; Pope *et al.,* 2012). In our model, this would mainly influence the amount of N_2_ that can be dissolved in them. If volume change also influences the effective hydrothermal circulation rate, it may play a more important role, as can be appreciated from Fig. 6 (simulations including volume change are also presented in Appendix B).

We do not consider the potential sequestration of N species in ice caps or loss to space. The quantity of nitrogen trapped in ice on the modern Earth is estimated to be ∼2.6 × 10^11^ kg (Wolff, 1995), which is orders of magnitude less than other reservoirs. As N_2_ is a relatively heavy gas, the terrestrial loss rate due to sputtering and thermal escape is also assumed to be negligible with some caveats; see for example the work of Shizgal and Arkos (1996) or Lammer *et al.* (2011).

Another effect that we did not model is the influence of the atmosphere on surface temperature. This represents a coupled feedback which may be important when considering specific planetary configurations. However, tripling the amount of N_2_ in the atmosphere is estimated to give a 3–8 K increase in mean surface temperature, which would negligibly affect Henry's law constants, adsorption isotherms (Goldblatt *et al.,* 2009), or erosion rates (Bland and Rolls, 1998). Thus, variations in atmospheric N_2_ content could create oscillatory dynamics in temperature and nitrogen cycling too fine to be discovered by this simple model.

This model assumes a constant marine sediment mass of fixed composition and fixed adsorption capacity. The uncertainty in this parameter may be large (see for example the discussion in Veizer [1973]), and sediment volume is generally related to the age of underlying oceanic crust (Hay *et al.,* 1988). In fact, the seafloor is very heterogeneously covered in sediments of varying ages, depths, and composition, and marine sedimentation is related to continental growth and biological activity (McLennan and Taylor, 1983). It is possible there was relatively little sediment present in the earliest oceans, and the facility of subducting adsorbed nitrogen was consequently lower. The composition and adsorption capacity of global marine sediments over deep time is as yet poorly constrained.

Several other potentially important contributions were also not considered in this study. These were neglected in order to keep the model tractable, but as they become more constrained, their impact on global cycling should be assessed. Here, we provide order-of-magnitude estimates and possible trends based on first-order considerations.

Metamorphism in the continental crust is a direct source of N_2_ for the atmosphere. Assuming 75% of nitrogen is releasable by metamorphism (Boudou *et al.,* 2008), and this metamorphism occurs over a billion-year timescale, this could provide a return flux to the atmosphere on the order of 10^9^ kg N yr^−1^, which is smaller than other modeled fluxes, which are on the order of 10^10^ to 10^11^ kg N yr^−1^. However, if the average age of metamorphosed rocks is much shorter (*e.g.,* 100 Ma), this flux becomes greater by an order of magnitude and will play a larger role, with the net effect of increasing pN_2_ and decreasing the amount of N sequestered in the continental crust.

Another potential source of N_2_ back to the atmosphere is reaction of odd nitrogen species in the oceans. Abiotic reactions of dissolved odd nitrogen species are suspected to play an important role in the nitrogen cycle (Zhu-Barker *et al.,* 2015), and it seems likely that there would have been a rich variety of aqueous chemistry in the primitive oceans that would have impacted the dynamics of these species. Acknowledging that we do not differentiate between HNO_2_ and HNO_3_ for NO*_x_* species, or for example between ammonia and hydrazine for NH*_x_* species, we can nevertheless place upper limits on the rates that may affect the principle reactive species.

An especially potentially important reaction in this regard is that of aqueous ammonium nitrite to give N_2_ and H_2_O (Nguyen *et al.,* 2003). Solving the rate equation for a modern ocean pH value (∼8) and 25°C gives a rate of N_2_ evolution by this mechanism of ∼4.5 × 10^−9^ *M*^−2^ yr^−1^. Assuming typical concentrations estimated here gives annual N_2_ return fluxes from the oceans to the atmosphere of ∼5.2 × 10^2^ to 2.6 × 10^12^ kg N yr^−1^, depending on the ratios of reactant species. While we expect HNO_2_ to be less abundant than HNO_3_, and thus for these to be upper boundaries on return rates, this does point to the potential extreme importance of low-temperature aqueous phase chemistry to an overall abiotic N-cycle.

There are undoubtedly many other potential reactions that deserve exploration, such as photochemistry in surface waters and redox disproportionation with dissolved transition metal species, such as iron (II) and (III) (Zhu-Barker *et al.,* 2015). Such considerations are seriously hampered by the complexities of aqueous geochemistry and uncertainties regarding the abundance of dissolved metal ions and carbonate species (*e.g.,* which influence pH and could precipitate iron (II) as siderite) in the primitive oceans, as well as uncertainties in the global average ocean pH and temperature, or how these values may have changed over time.

Finally, the nature of mantle mixing and differentiating between degassing of primordial nitrogen versus degassing of subducted nitrogen is beyond the scope of this model. Tracking of isotopic differences could be added to this model to add to the ways it can be compared to observations. This may also help understand partitioning between different types of degassing rates.

### 4.2. Implications for Earth

In both the low and high abiotic atmospheric fixation rate scenarios, a key feature of our model is the large NH*_x_* reservoir in the oceans. None of the processes modeled here are able to cycle it back to the mantle fast enough to match values similar to that of the present-day Earth. A potential implication is that the net effect of biotic processes is to use this easily available nitrogen source for biomass production, increasing the flux of nitrogen transported to the continental crust and mantle through enhanced sedimentation. If the low abiotic fixation rate case is more representative, life may be the missing conduit that bypasses the atmospheric bottleneck (*i.e.,* the fact that sources to the atmospheric nitrogen budget are much greater than its sinks) and efficiently redistributes N_2_ back into global reservoirs.

Another observation can be made that relates to measurements that have estimated total atmospheric pressure to have been less than 0.5 PAL 2.7 billion years ago (Som *et al.,* 2016). It is interesting to note that, for the high abiotic atmospheric fixation rate case, the net effect of life would be to add N_2_ back to the atmosphere, and denitrification would have to become a major flux later than 2.7 Ga. On the other hand, for the low abiotic atmospheric fixation rate case, the net effect of life would be to draw N_2_ down from the atmosphere, and in that case biotic N-fixation would have to start earlier (and possibly considerably earlier) than 2.7 Ga such that it would match paleo-pressure estimates. Note, however, that Marty *et al.* (2013) estimated pN_2_ to be similar to present-day value 3–3.5 billion years ago, so either large fluctuations have occurred over geological time or better constraints on pN_2_ and total atmospheric pressure as a function of time are required.

### 4.3. Implications for remote sensing

The main difference between this and previous models is the explicit treatment of the oceans and different speciations of nitrogen. In agreement with the work of Stüeken *et al.* (2016), we find that the overall trend of an abiotic planet with geological processes similar to those of Earth is long-term increase in pN_2_. However, depending on the lightning-induced abiotic fixation rate, the long-term evolution is different. For high abiotic fixation rates, the atmospheric N_2_ variation is limited to 10–20% of the atmospheric mass, and therefore it may not lead to any observable effect within error bars, as pointed out by Zhang and Zindler (1993); whereas for low abiotic fixation rates, there is a significant buildup of nitrogen in the atmosphere. In both cases, however, there is a large amount of nitrogen stored in the oceans as soluble NH_*x*_.

This study suggests that, if little nitrogen is observed in an atmosphere relative to an expected bulk content (for instance using N_2_-N_2_ collisions [Schwieterman *et al.,* 2015]), and loss to space can be excluded, the bottleneck to global cycling is located in another reservoir besides the atmosphere. For a planet with geophysical parameters similar to those of Earth, this bottleneck is likely the flux back to the mantle (*i.e.,* due to the lack of plate tectonics, or poor adsorption/subduction efficiency). In contrast, if a large fraction of the expected nitrogen budget is found in the atmosphere, our model suggests that it is due to a low atmospheric abiotic fixation rate. If atmospheric chemistry does not make that explanation probable, then it may suggest particularly efficient transport of nitrogen from the crust to a relatively oxidized mantle.

Another implication is that large variations in nitrogen content of a given reservoir do not necessarily imply a qualitative change in processes (*e.g.,* the onset of plate tectonics, an oxygenation event). We found that the collapse of initial conditions to the model steady state takes a few hundred million years and may be non-monotonic, which comes from the fact that arbitrary initial conditions may be far from the long-term evolutionary trend. This also implies that, in cases where the early evolution is important, the choice of initial conditions plays an important role in the expected distribution of nitrogen species among planetary reservoirs.

## 5. Conclusions

In the absence of life, nitrogen is still actively redistributed among reservoirs by abiological planetary dynamics and processes. Understanding what controls this cycling can therefore be used as a remote observable for planetary dynamics and could help identify inhabited planets. In particular, understanding what controls the global fraction of nitrogen degassed to the atmosphere (*e.g.,* geophysical and geochemical vs. biological processes) can help remote sensing observations make inferences about surface and interior processes.

This model suggests that the timescales involved in the evolution of N cycling allow a planet's geodynamical system to closely track predictable steady states, and it suggests that the long-term evolution of N cycling is governed by variations in the steady state due to environmental parameters. We have shown that changes in dynamical parameters (such as ocean hydrothermal circulation or mantle mixing rates, subduction rate, mantle and atmosphere redox state, etc.) lead to direct changes in nitrogen distribution and therefore that remote sensing of this distribution (as seen in atmospheric N_2_ content) can, in principle, be related to these parameters, as also suggested by Stüeken *et al.* (2016).

Assuming that the bulk nitrogen content of a planet can be estimated (from planetary formation considerations and stellar properties), and the age of a planetary system can be obtained from observation of its host star, the amount of N present in a planet's atmosphere could in principle be used to draw inferences about surface and interior processes, such as ocean volume, mantle mixing rate and redox state, the existence of plate tectonics, and the presence of life.

For example, if most of an abiotic planet's nitrogen is in its atmosphere, this implies there is no efficient transport to the interior, which can be due to lack of plate tectonics or no hydrologic cycle. Any amount below this can be used to estimate the magnitude of cycling between surface and interior. This model is obviously very simple but benefits from the fact that N_2_ is remarkably chemically inert and its chemistry therefore relatively unaffected by the many nuances that would be extremely important for elements such as carbon or sulfur. There may be other elements or species not considered here whose dynamics are also, and possibly more, useful as indicators of biology on planets beyond Earth; however, N may be unique in this regard.

## Appendix A. Nitrogen Distribution

Samples of a large number of carbonaceous chondrite meteorites give a mean nitrogen content of 3180 ppm (McDonough and Sun, 1995), although many have lower abundances (Johnson and Goldblatt, 2015). Given that the mass of Earth is ∼6 × 10^24^ kg, this gives 0.7–1.9 × 10^19^ kg total nitrogen (Table 1). An approximate inventory of ∼10^19^ kg N is commonly cited (Capone *et al.,* 2006), though values of 2.0–3.3 × 10^20^ kg N have also been offered (Johnson and Goldblatt, 2015). The variability in these estimates largely reflects uncertainties in the nitrogen content of the core.

Deep reservoirs likely play host to the largest amount of Earth's nitrogen. Recent high-pressure studies indicate that minerals such as β-Fe_7_N_3_ are stable under deep Earth conditions and that the core could contain Fe_*x*_N_*y*_ phases accounting for a massive 9.8 × 10^18^ kg N (Minobe *et al.,* 2015), or ∼50% of a proposed chondrite-derived inventory.

The average concentration of nitrogen in mantle-derived rocks has been estimated as between 0.2–2.1 ppm (Johnson and Goldblatt, 2015) and 2.8 + 1.0/-0.8 ppm (Marty, 1995). Given that the volume of the mantle is ∼1.2 × 10^7^ km^3^, and its density is ∼4000 kg m^−3^, there is expected to be 3.4 × 10^18^ kg N in the mantle, or approximately 20% of a proposed chondrite-derived inventory. There is also considerable uncertainty in this value; for comparison, Zhang and Zindler (1993) estimated that the amount of N_2_ in the present mantle is 2.5 × 10^19^ mol (7 × 10^17^ kg N). Several authors have estimated that the vast majority of this nitrogen is at the −3 or 0 oxidation state (Li and Keppler, 2014; Mikhail and Sverjensky, 2014). On the other hand, using N-Ar geochemistry, Johnson and Goldblatt (2015) showed that the present-day mantle likely contains 7 ± 4 PAL nitrogen (2.8 ± 1.6 × 10^19^ kg N).

Earth's surface reservoirs, including the atmosphere, crust, oceans, and sedimentary rocks, are more amenable to direct measurement, and it is estimated that together they contain 5.9 × 10^18^ kg N (Johnson and Goldblatt, 2015). There is estimated to be 4 × 10^18^ kg N in the atmosphere (*e.g.,* Johnson and Goldblatt [2015] gave a value of 3.87 × 10^18^ kg). Together, the atmosphere and mantle account for ∼15% of a proposed chondrite-derived inventory. It is estimated there is an additional 1.9 × 10^18^ kg N in the crust, oceans, and sedimentary rocks (Johnson and Goldblatt, 2015), accounting for an additional 5% of the chondritic estimate. It is worth noting, for reference, that if all the nitrogen in contemporary surface reservoirs was dissolved in oceans of the current volume, a solution ∼0.2 *M* in single N-atom containing species (*e.g.,* ammonium, nitrite or nitrate) would be obtained (Schwartz, 1981).

## Appendix B. Appendix Figures

### Appendix B.1. Subduction rate

**FIG. B1.**
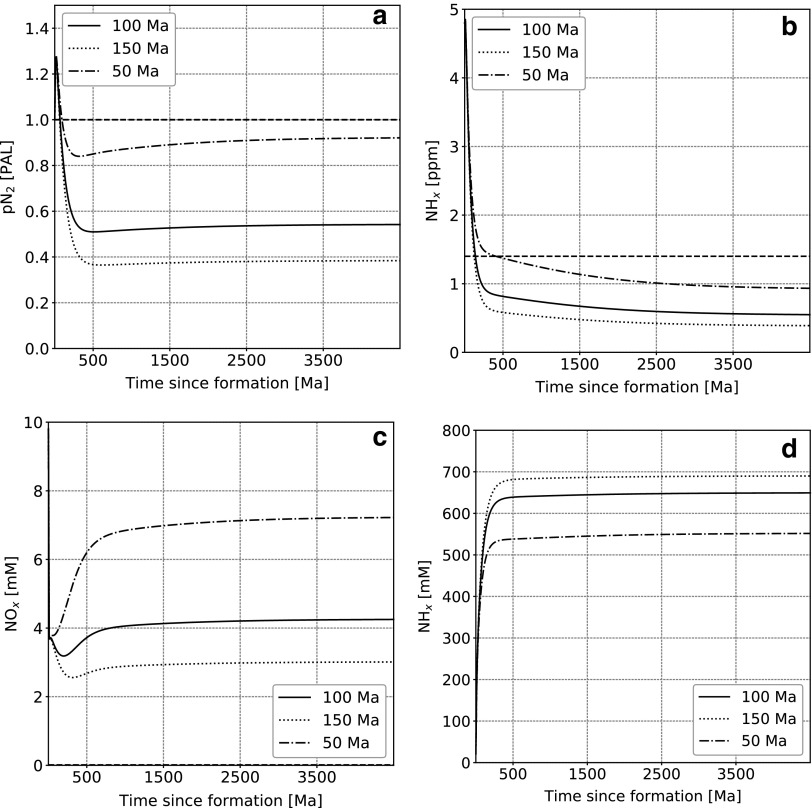
Nitrogen content as a function of time in the main model reservoirs for different average oceanic crust subduction rates. (**a**) Atmospheric N_2_, (**b**) lower mantle (NH_*x*_), oceanic NO*_x_* (**c**) and NH*_x_* (**d**). Dashed lines represent present-day values. For oceanic NO*_x_* and NH*_x_* these values are 10^−2^ and 3 × 10^−4^ m*M,* respectively, and therefore do not appear on the figures.

We varied the subduction rate by a factor of 3 to sample a plausible range of values (Fig. B1). For the fastest case (complete oceanic crust recycling in 50 Ma), the atmospheric N_2_ content is maximal, which is associated with a decrease of NH*_x_* in the ocean, as more is adsorbed and subducted. In contrast, for the largest value (complete cycling in 150 Ma), a larger fraction of nitrogen remains in the oceans as NH_*x*_. A fast cycling rate also favors transport of nitrogen to the interior, which induces stronger degassing, as can be seen in the ocean NO*_x_* content.

### Appendix B.2. Ocean volume

**FIG. B2.**
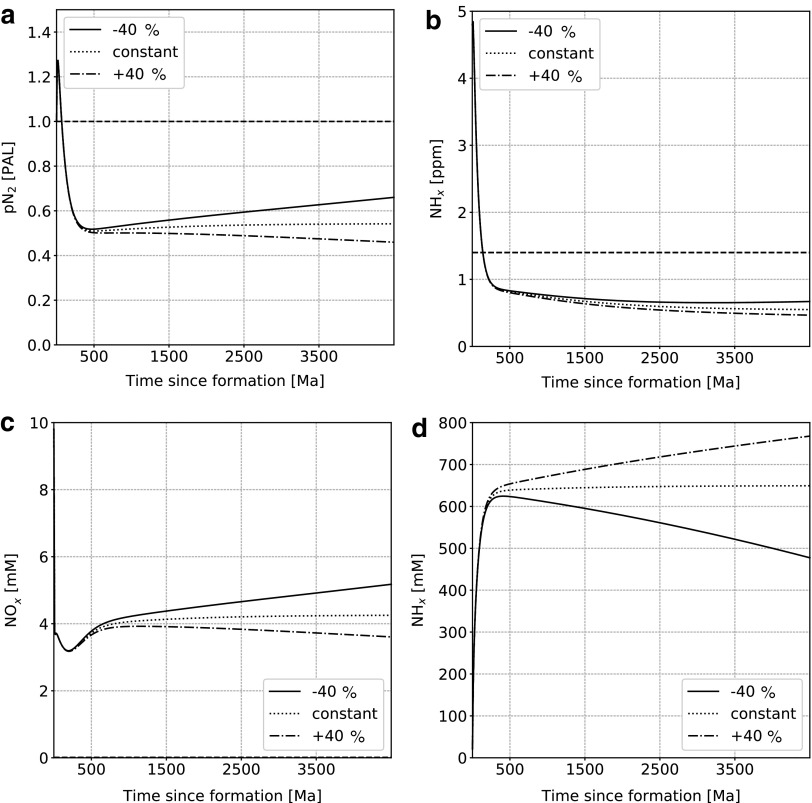
Nitrogen content as a function of time in the main model reservoirs for different ocean volume evolution scenarios (values are given in percent change since formation). (**a**) Atmospheric N_2_, (**b**) lower mantle (NH_*x*_), oceanic NO*_x_* (**c**) and NH*_x_* (**d**). Dashed lines represent present-day values. For oceanic NO*_x_* and NH_*x*_, these values are 10^−2^ and 3 × 10^−4^ m*M,* respectively, and therefore do not appear on the figures.

The change in ocean volume as a function of time is uncertain. We therefore tested scenarios with a linear change of volume over time (Fig. B2). An increase in volume allows more nitrogen to be dissolved and thus results in a lower atmospheric pN_2_. This leads to more nitrogen being cycled through hydrothermal vents and, thus, a higher NH*_x_* content. In contrast, as no additional source of NO*_x_* is added, its concentration is slightly reduced. The influence on the lower mantle is also minimal.

### Appendix B.3. Evolution with a small nitrogen budget

**FIG. B3.**
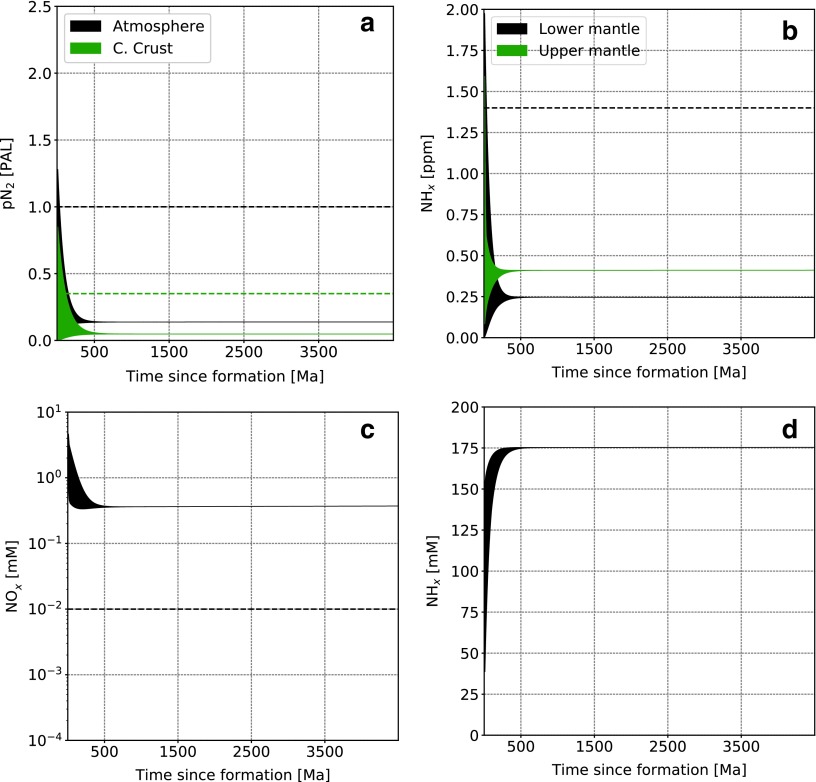
Nitrogen content as a function of time in the main model reservoirs. (**a**) Atmospheric N_2_, (**b**) lower mantle (NH_*x*_), oceanic NO*_x_* (**c**) and NH*_x_* (**d**). Dashed lines represent present-day values. For upper mantle and oceanic NH_*x*_, these values are 3.5 ppm and 3 × 10^−4^ m*M,* respectively, and therefore do not appear on the figures.

**FIG. B4.**
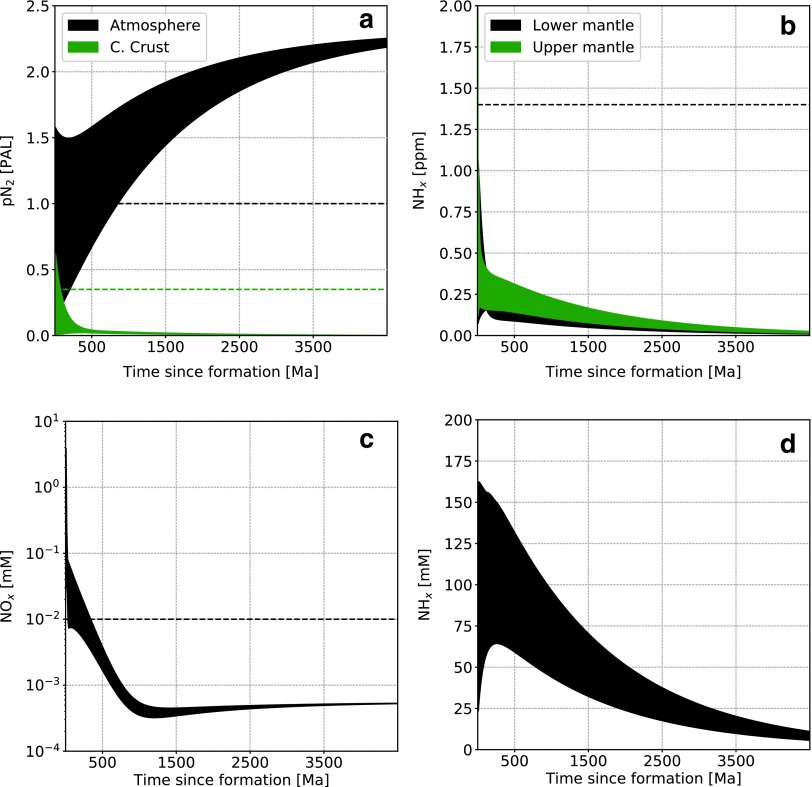
Nitrogen content as a function of time in the main model reservoirs. (**a**) Atmospheric N_2_, (**b**) lower mantle (NH_*x*_), oceanic NO*_x_* (**c**) and NH*_x_* (**d**). Dashed lines represent present-day values. For upper mantle and oceanic NH_*x*_, these values are 3.5 ppm and 3 × 10^−4^ m*M,* respectively, and therefore do not appear on the figures.

As discussed in the work of Johnson and Goldblatt (2015), the bulk silicate Earth nitrogen budget is uncertain, and the 3.4 × 10^19^ kg N value that we used is a high-end estimate. Here we present typical evolution curves for a global budget of 9.3 × 10^18^ kg N. Figure B3 is for a high abiotic atmospheric fixation rate and corresponds to Fig. 3. Figure B4 is for a low abiotic atmospheric fixation rate and corresponds to Fig. 4. The qualitative behavior is very close to the case with the larger bulk silicate nitrogen budget, but with all reservoirs being reduced by a similar ratio.

## Abbreviations Used

MORB: mid-ocean ridge basalt
PAL: present atmospheric levels
